# Zinc Oxide and Magnesium-Doped Zinc Oxide Nanoparticles Ameliorate Murine Chronic Toxoplasmosis

**DOI:** 10.3390/ph17010113

**Published:** 2024-01-15

**Authors:** Mohamed H. Sarhan, Shatha G. Felemban, Walla Alelwani, Hesham M. Sharaf, Yasmin A. Abd El-Latif, Elsayed Elgazzar, Ahmad M. Kandil, Guillermo Tellez-Isaias, Aya A. Mohamed

**Affiliations:** 1 Microbiology Section, Basic Medical Sciences Department, College of Medicine, Shaqra University, Shaqra 11961, Saudi Arabia; 2 Medical Parasitology Department, Faculty of Medicine, Zagazig University, Zagazig 44519, Egypt; 3 Medical Laboratory Science Department, Fakeeh College for Medical Sciences, Jeddah 21461, Saudi Arabia; sfelemban@fcms.edu.sa; 4 Department of Biochemistry, College of Science, University of Jeddah, Jeddah 23890, Saudi Arabia; welwani@uj.edu.sa; 5 Zoology Department, Faculty of Science, Zagazig University, Zagazig 44519, Egypt; sharafhesham421960@gmail.com (H.M.S.); yasminahmed@zu.edu.eg (Y.A.A.E.-L.); ayaali@zu.edu.eg (A.A.M.); 6 Physics Department, Faculty of Science, Suez Canal University, Ismailia 41522, Egypt; 7 Pathology Department, Faculty of Medicine, Al-Azhar University, Cairo 11651, Egypt; dr.ak2009@yahoo.com; 8 Department of Poultry Science, Division of Agriculture, University of Arkansas, Fayetteville, AR 72701, USA

**Keywords:** brain, CD31, chronic toxoplasmosis, ME49 strain, Mg-doped ZnO, nanoparticles, P53, *Toxoplasma*, ZnO

## Abstract

*Toxoplasma gondii* causes a global parasitic disease. Therapeutic options for eradicating toxoplasmosis are limited. In this study, ZnO and Mg-doped ZnO NPs were prepared, and their structural and morphological chrematistics were investigated. The XRD pattern revealed that Mg-doped ZnO NPs have weak crystallinity and a small crystallite size. FTIR and XPS analyses confirmed the integration of Mg ions into the ZnO framework, producing the high-purity Mg-doped ZnO nanocomposite. TEM micrographs determined the particle size of un-doped ZnO in the range of 29 nm, reduced to 23 nm with Mg^2+^ replacements. ZnO and Mg-doped ZnO NPs significantly decreased the number of brain cysts (*p* < 0.05) by 29.30% and 35.08%, respectively, compared to the infected untreated group. The administration of ZnO and Mg-doped ZnO NPs revealed a marked histopathological improvement in the brain, liver, and spleen. Furthermore, ZnO and Mg-doped ZnO NPs reduced P53 expression in the cerebral tissue while inducing CD31 expression, which indicated a protective effect against the infection-induced apoptosis and the restoration of balance between free radicals and antioxidant defense activity. In conclusion, the study proved these nanoparticles have antiparasitic, antiapoptotic, and angiogenetic effects. Being nontoxic compounds, these nanoparticles could be promising adjuvants in treating chronic toxoplasmosis.

## 1. Introduction

*Toxoplasma gondii*, a member of *Apicomplexa*, is an intracellular parasitic protozoan that infects approximately one in three people worldwide, causing a devastating disease called toxoplasmosis that threatens not only human beings but also bird and animal populations [1,2,3]. The feline is the only known definitive host of *T. gondii* [4]. The infection is transmitted to warm-blooded vertebrates through food or water contaminated with feline feces or undercooked meat containing tissue cysts [5]. Chronic toxoplasmosis infection is usually asymptomatic in healthy people with an exemplary immune system and, at worst, causes vague flu-like symptoms such as a mild fever and headache [6]. However, in immunocompromised patients, it could develop severe clinical consequences that may be fatal, like toxoplasmic encephalitis [1,2]. Furthermore, chronic infection with *T. gondii* may lead to several mental health issues, such as depression and schizophrenia [7].

Moreover, *Toxoplasma* infection regulates many cellular pathways to support its survival and proliferation. One of the host pathways impacted by *T. gondii* intracellular invasion is the genetically programmed cell death process known as apoptosis. Plenty of proteins regulate the process of apoptosis, such as P53 [8]. Although the activity of P53 is unnecessary for normal cell development, its critical role in parasitic infections has been mentioned in some previous studies that make sense as the activation of P53 is associated with pathological changes in the cell such as cell cycle arrest, oxidative stress, and DNA damage [8,9]. Another pathway impacted by toxoplasmosis infection is called angiogenesis or neovascularization. Angiogenesis represents the growth of new blood vessels that sprout from existing vasculature and involves differentiation, growth, and migration of the endothelial cells lining the blood vessels [10]. Platelet endothelial cell adhesion molecule-1 (PECAM-1 or CD31) is one of the most widely utilized biomarkers of endothelial differentiation, as it participates in the interaction between neighboring endothelial cells that regulates blood flow by rearranging the cell’s outer boundary [11].

Therapeutic options for eradicating toxoplasmosis are limited, along with their poor efficacy due to their restricted ability to penetrate the brain and destructive side effects [12,13,14]. For a long time, the pyrimethamine (PYR) and sulfadiazine (SDZ) combination has been vigorously considered the ideal treatment against toxoplasmosis as it prevents the synthesis of folic acid, inhibiting the proliferation of parasites [2,12,13]. Unfortunately, the application of this medication has been reported to have severe side effects, including folic acid deficiency due to the suppression of folate production, hematological toxicity, teratogenic complications, cardiac arrhythmia, and hypersensitivity reactions [1,15]. Furthermore, until now, there has been no suitable and effective anti-*toxoplasma* vaccine ready-made to control this opportunistic parasite [16]. Hence, novel drugs with limited negative consequences and high effectiveness are needed. 

Recently, the utilization of nanotechnology in the treatment of different diseases, particularly infectious ones, has grown significantly [17]. Metal oxide-based binary and ternary nanoparticles (NPs) have gained great attention because of their remarkable applications in medicine, catalysis, wound healing, pharmaceuticals, biosensors, and agriculture. Nearly 1800 commercial healthcare nanomaterial products like skin creams and band-aids are already available on the market [18,19]. To date, tremendous techniques have been employed for preparing nanomaterials [20,21]. Chemical techniques, in particular, are scalable, affordable, and controllable options. Moreover, the metal oxide NPs can generate reactive oxygen species (ROS), eliminating infectious parasites such as *T. gondii* [22]. Overall, nonantibiotics have emerged as a promising medication for combating pathogenic bacteria and fungi without negatively affecting public health or the environment [21,23].

Zinc oxide (ZnO) NPs are considered the most promising metal oxide due to their ability to pass throughout the body and penetrate the brain tissue [24], and they could also accumulate in tissues, providing an effective medium to devastate *T. gondii* tissue cysts. The antiparasitic activity of ZnO NPs has been previously described against many infectious diseases, including leishmaniasis [25,26], giardiasis [27], coccidiosis [28], schistosomiasis [29], and even acute toxoplasmosis [30]. Metal ions, such as aluminum, can be doped into ZnO NPs to enhance their anti-microbial properties [22]. The divalent magnesium ion (Mg^2+^) is a significant dopant element because its atomic radius with Zn^2+^ is equivalent, inducing surface trapping levels and defects in the band structure of the ZnO lattice and, consequently, adjusting the optical band gap [31]. Indeed, Mg-doped ZnO NPs demonstrated ROS generation and Zn^2+^ release and enhanced their antibacterial properties [32]. Furthermore, Mg-doped ZnO NPs have no cytotoxic effect, as previously concluded by Hamdy et al. [33].

Thus, to fulfill the demand for novel anti-toxoplasmosis drugs, ZnO NPs and Mg-doped ZnO NPs were synthesized and studied using several techniques to demonstrate their properties and characteristics. Then, the antiparasitic potential activity of these NPs was tested against chronic toxoplasmosis induced by the cystogenic strain (ME49) of the coccidian parasite *T. gondii* in experimentally infected mice. Hence, the assessment was based on parasitological estimation, histopathological examination, and immunohistochemical evaluation of P53 and CD31 expressions.

## 2. Results

### 2.1. Crystal Structure, Functional Group, Chemical Valence, and Morphological Analysis

#### 2.1.1. XRD Analysis

**Figure 1:**
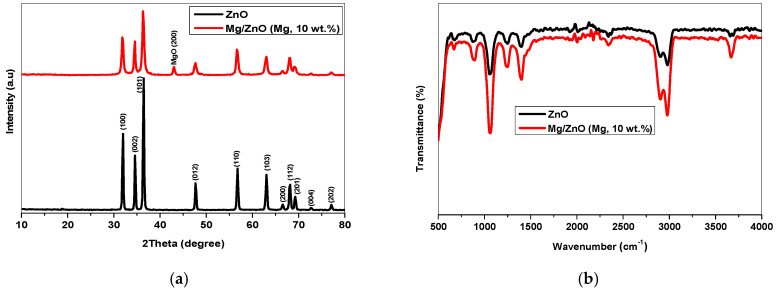
(**a**) XRD pattern and (**b**) FTIR of un-doped ZnO and Mg-doped ZnO NPs.

The crystal structure of un-doped ZnO and Mg-doped ZnO nanostructures was discussed from XRD through 2θ, ranging from 10 to 80°. Figure 1a shows a ZnO pattern of sharp, intense peaks revealing a high degree of crystallinity and large crystal size. The diffraction peaks located at 2θ = 31.99°, 34.59°, 36.43°, 47.79°, 56.81°, 63.09°, 66.59°, 68.19°, 69.44°, 72.70°, and 77.13° according to the reflection planes (100), (002), (101), (012), (110), (103), (200), (112), (201), (004), and (202) are matched with the JCPDS card No. 05-0669 of pure ZnO. For further crystallographic analysis, the XRD parameters comprising crystalline size (D), dislocation density (δ), strain (ε), and crystallinity degree (X_c_) were estimated from the most intense three peaks of planes (100), (002), and (101) using the following equations:

D = Kλ/βcosθ, ε = βcosθ/4, δ = 1/D^2^, and X_c_ = 0.24/β

The obtained results are summarized in Table 1.

#### 2.1.2. FTIR Analysis

The molecular vibration, chemical bonding, and functional group of ZnO and Mg-doped ZnO NPs were investigated using FTIR spectra through a wavenumber range of 500–4000 cm^−1^. As demonstrated in Figure 1b, the FTIR spectra of the prepared samples have a similar behavior; however, the doped sample shows strong wide bands and high intensity. 

#### 2.1.3. XPS Analysis

**Figure 2:**
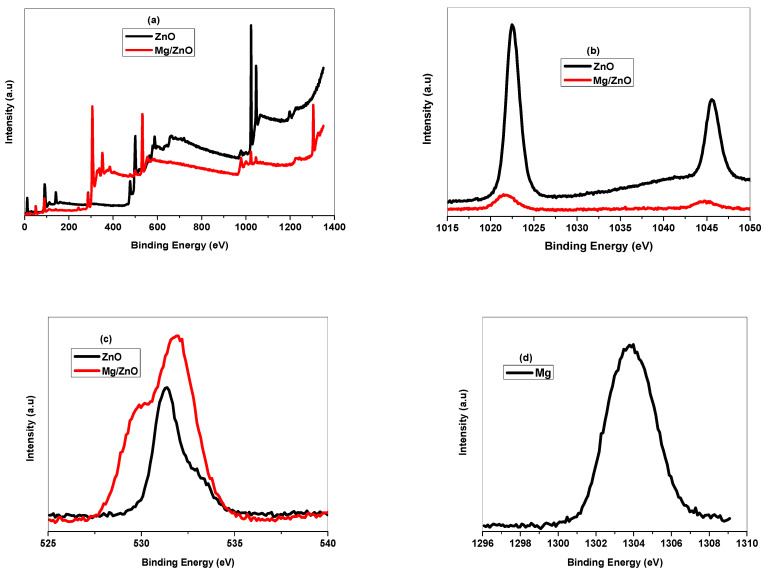
(**a**–**d**): XPS spectrum of un-doped ZnO and Mg-doped ZnO NPs.

The surface composition, chemical valence, and electronic states of the nanoparticles were analyzed by using XPS (Figure 2a,d). As seen in Figure 2a, the peaks attributed to Zn, O, and C elements exist in the survey spectrum without undesirable peaks. The Zn 2p spectrum depicts two peaks: 2p3/2 and 2p1/2 binding energies appear at 1022.62 eV and 1045.56 eV, assigned to Zn^2+^ in the host ZnO lattice (Figure 2b). Figure 2c shows the splitting of O 1s in the spinel structure into two signals located at 530.429 and 531.945 eV, corresponding to one peak at 531.356 eV in ZnO. Figure 2d displays the chemical valence of the Mg 1 s level at 1304.35 eV, revealing the presence of metallic magnesium.

#### 2.1.4. TEM Analysis

**Figure 3:**
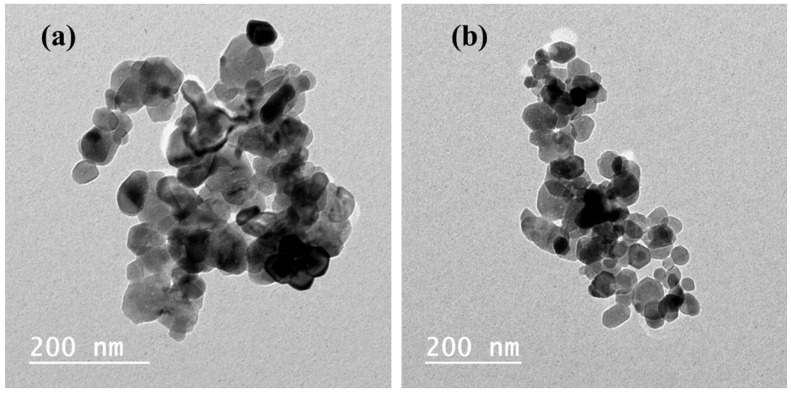
TEM micrographs of un-doped (**a**) ZnO and (**b**) Mg-doped ZnO NPs.

The shape and average size of NPs were visualized from TEM micrographs. Figure 3a shows the particles of ZnO in hexagonal-like shapes with a mean size of 29 ± 3.86 nm. Figure 3b shows the particles of Mg-doped ZnO at a small size of 23 ± 5.82 nm with high density.

### 2.2. Parasitological Assessment

#### 2.2.1. Parasitic Burden Assessment 

The potential load of chronic toxoplasmosis was calculated by counting the total number of tissue cysts detected in 10 µL of brain homogenate from each mouse in all infected groups. The main tissue cyst numbers are summarized in Table 2. As illustrated, ZnO and Mg-doped ZnO NPs significantly decreased the number of brain cysts (*p* < 0.05) by 29.30% and 35.08%, respectively, compared to the infected untreated group. Moreover, there is a significant difference (*p* < 0.05) between the groups treated with ZnO and Mg-doped ZnO NPs.

#### 2.2.2. Histopathological Assessment 

For assessment of the therapeutic effects of ZnO and Mg-doped ZnO NPs on chronic toxoplasmosis, histopathological sections of the brain, liver, and spleen from the different experimental groups were examined.

- Brain

**Figure 4:**
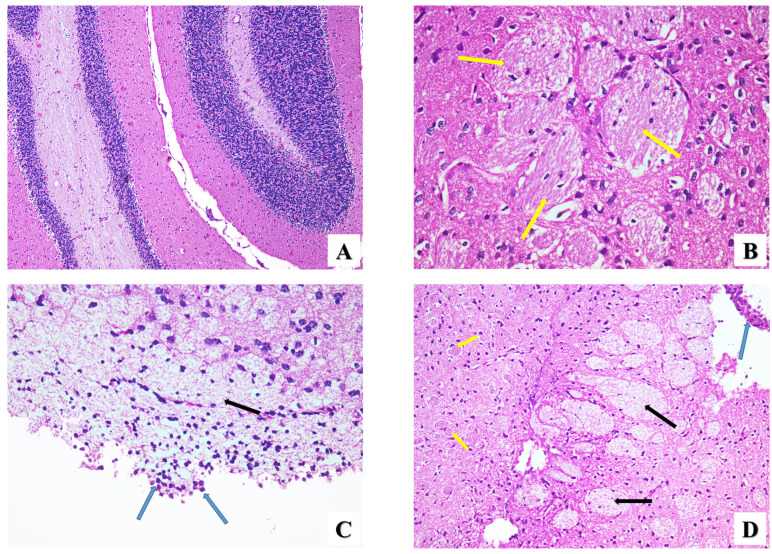

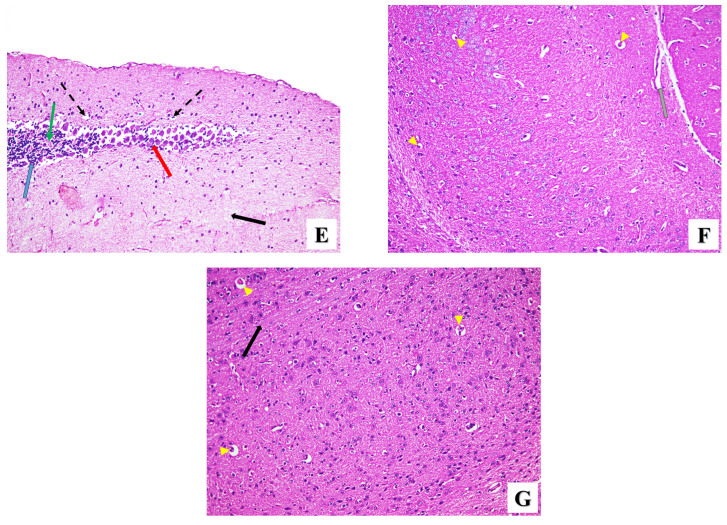
Microscopic histopathological representative images showing the histopathological changes in brain tissues of all groups in this study. (**A**) Healthy brain showing normal brain architecture (H&E ×100). (**B**) Histopathological changes in GII showing severe gliosis (yellow arrows) (H&E ×400). (**C**) Brain section of the infected untreated group showing brain edema (black arrow) and inflammatory cells (blue arrows) (H&E ×400). (**D**) Histopathological changes in GIII showing severe brain edema (black arrows), moderate gliosis (yellow arrows), and few inflammatory cells (blue arrow) (H&E ×200). (**E**) Brain section of GIII showing brain edema (black arrow), accumulation of red neurons (red arrow), degenerated neural cells (black dashed arrows), hemorrhage (green arrow), and inflammatory cells (blue arrow) (H&E ×200). (**F**) GIV group showing degenerated tissue cysts (yellow arrowheads) and dilated blood vessels (gray arrow) (H&E ×200). (**G**) GV group showing degenerated tissue cysts (yellow arrowheads) and mild brain edema (black arrow) (H&E ×200).

Brain sections from all experimental groups were histologically investigated to evaluate the pathogenesis of the brain tissue. When the brain cortex of the healthy group (GI) was examined, it was found to have a typical morphology comprising a combination of pyramidal cells and neuroglial cells (Figure 4A). Furthermore, capillaries appeared completely normal. In contrast, brain sections of infected mice (GII) clearly showed tissue cysts of *T. gondii* associated with severe gliosis and brain edema (Figure 4B). Encephalitis and neuronal degeneration were also observed (Figure 4C). On the other hand, brain sections of infected mice treated with PYR+SDZ (GIII) showed brain edema accompanied by a lower degree of brain gliosis compared to the infected brain sections and encephalitis with mononuclear cell infiltration (Figure 4D). Numerous red neurons, which reveal acute injury in neurons and subsequent apoptosis, are shown in Figure 4E. After treatment with ZnO NPs (GIV), the histopathological examination of the brain sections manifested a kind of near-normal histological tissue with degenerated tissue cysts and mildly dilated blood vessels (Figure 4F). Furthermore, the brain sections of infected mice treated with Mg-doped ZnO NPs (GV) showed degenerated tissue cysts and mild brain edema without any inflammatory cells (Figure 4G). However, the combination of PYR and SDZ did not result in a substantial change in the inflammation severity of infected brain tissue; intraperitoneal injection of ZnO and Mg-doped ZnO NPs significantly reduced (*p* < 0.05) the inflammatory score (IS) by 72% and 80%, respectively (Table 3), without a significant difference in between.

- Liver

**Figure 5:**
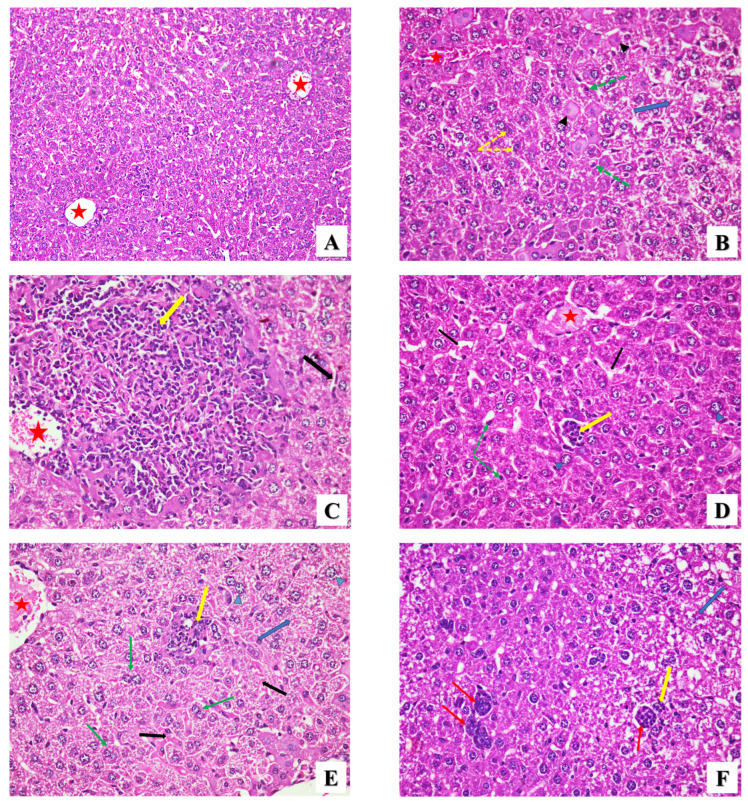
Microscopic histopathological representative images showing the histopathological changes in liver tissues of all groups in this study. (**A**) Normal health group (GI) showing uniform hepatocytes with eosinophilic cytoplasm around the central veins (red stars) and normal sinusoids (H&E ×400). (**B**) Liver sections of the infected untreated group (GII) showing hydropic degeneration (blue arrow), acidophilic cytoplasm (black arrowheads), pyknotic nuclei (green dashed arrows), karyorrhexis (yellow dashed arrows), and vascular congestion (red star) (H&E ×400). (**C**) Infected group showing severe periportal inflammatory cellular infiltration (yellow arrow), vascular congestion (red star), and sinusoidal dilation (black arrow) (H&E ×400). (**D**) Infected group treated with PYR+SDZ combination (GIII) showing ballooning of some hepatocytes (blue arrowheads), mononuclear inflammatory cells (yellow arrow), pyknotic nuclei (green dashed arrows), congestion of central vein (red star), and sinusoidal dilation (black arrows) (H&E ×400). (**E**) ZnO NP-treated group (GIV) showing hydropic degeneration (blue arrow), ballooning of some hepatocytes (blue arrowheads), binucleated hepatocytes (green arrows), lymphatic inflammatory cells (yellow arrow), congested central vein (red star), and sinusoidal dilation (black arrows) (H&E ×400). (**F**) Administration of Mg-doped ZnO NPs (GV) showing elongated and circular tissue cysts (red arrows) with some inflammatory cells (yellow arrow) and hydropic degeneration (blue arrow) (H&E ×400).

High magnifications of different fields of liver sections from uninfected mice (GI) showed uniform hepatocytes with eosinophilic cytoplasm and rounded, prominent vesicular nuclei arranged around the central vein and normal sinusoids between the hepatic cellular cords (Figure 5A). Inversely, hepatocytes in liver sections of GII demonstrated severe histopathological necrotic changes such as hydropic degeneration, ballooning, acidophilic cytoplasm, pyknotic nuclei, and karyorrhexis (Figure 5B). Sinusoidal dilation and vascular congestion with marked periportal lymphocytic inflammatory cellular infiltration were also noted (Figure 5C). Moreover, the infected group treated with the PYR+SDZ combination (GIII) exhibited ballooning of some hepatocytes and necrotic degeneration of others with few mononuclear inflammatory cells, as well as marked congestion of the central veins and sinusoidal dilation (Figure 5D). For GIV treated with ZnO NPs, histopathological changes such as hydropic degeneration, lymphatic inflammatory cells, a congested central vein, and binucleated hepatocytes were observed (Figure 5E). The liver sections of GV demonstrated elongated tissue cysts and degenerated hepatocytes with few lymphatic inflammatory cells (Figure 5F). Moreover, the administration of ZnO and Mg-doped ZnO NPs significantly reduced (*p* < 0.05) the inflammation score (IS) in liver tissue by 65% and 72%, respectively (Table 3), without a significant difference in between.

- Spleen

**Figure 6:**
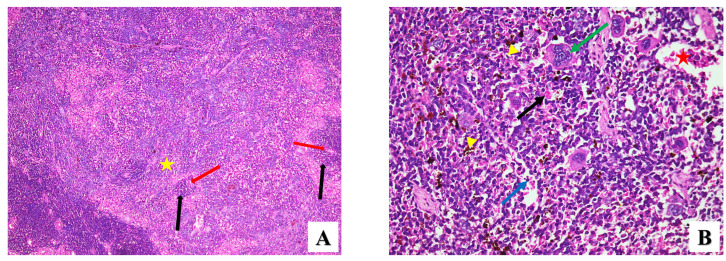

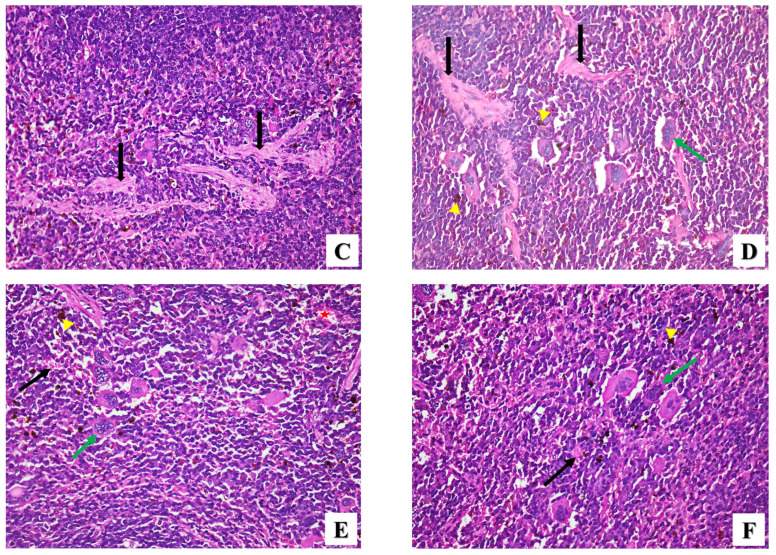
Microscopic histopathological representative images showing the histopathological changes in splenic tissues of all groups in this study. (**A**) Normal healthy group (GI) showing normal splenic tissue of red pulp (yellow star) and white pulp (black arrows) with follicular arteriole (red arrows) (H&E ×100). (**B**) Infected group (GII) demonstrating splenic architecture loss, macrophages infected by *T. gondii* (green arrow), plasma cells (blue arrow), extravasation of blood (black arrow) moderate deposition of hemosiderin pigment (yellow arrowheads), and congested blood vessels (red star) (H&E ×400). (**C**) Splenic section of infected group (GII) showing fibrinoid material in splenic trabeculae (black arrows) (H&E ×400). (**D**) Splenic section of GIII showing macrophages infected by *T. gondii* (green arrow), fibrinoid material (black arrows), and mild deposition of hemosiderin pigment (yellow arrowheads) (H&E ×400). (**E**) Splenic section of GIV showing macrophage (green arrow), mild deposition of hemosiderin pigment (yellow arrowhead), congested blood vessels (red star), and extravasation of blood (black arrow) (H&E ×400). (**F**) Splenic section of GV showing macrophage (green arrow), mild deposition of hemosiderin pigment (yellow arrowhead), and extravasation of blood (black arrow) (H&E ×400).

H&E-stained spleen sections for the healthy group (GI) showed normal histomorphology of the splenic pulps, the white and red pulps, and the capsule. The germinal central and follicular arterioles distinguished the white pulps from the red pulps, and the red pulps are composed of splenic cords and venous sinuses, which are detected by the presence of red blood cells (Figure 6A). On the contrary, lymphocytic depletion of splenic lymphoid follicles was noted in splenic sections of infected mice (GII), accompanied by numerous macrophages that were invaded by *T. gondii*, plasma cells, congested blood vessels, extravasation of blood, and moderate deposition of hemosiderin pigment (Figure 6B). Considerable fibrinoid material in splenic trabeculae was also seen (Figure 6C). Furthermore, splenic sections of GIII demonstrated the same histopathological changes, but more fibrinoid material and mildly deposited pigment were observed (Figure 6D). The splenic tissue of infected mice treated with ZnO NPs (GIV) showed normal histological architecture with congested blood vessels and had less hemosiderin pigment deposition than that seen in GIII (Figure 6E). Splenic sections of infected mice treated with Mg-doped ZnO NPs (GV) showed the same histopathological changes (Figure 6F). No significant reduction in IS was shown after treatment with ZnO or Mg-doped ZnO NPs in splenic tissue (Table 3).

#### 2.2.3. Immunohistochemical Assessment

**Figure 7:**
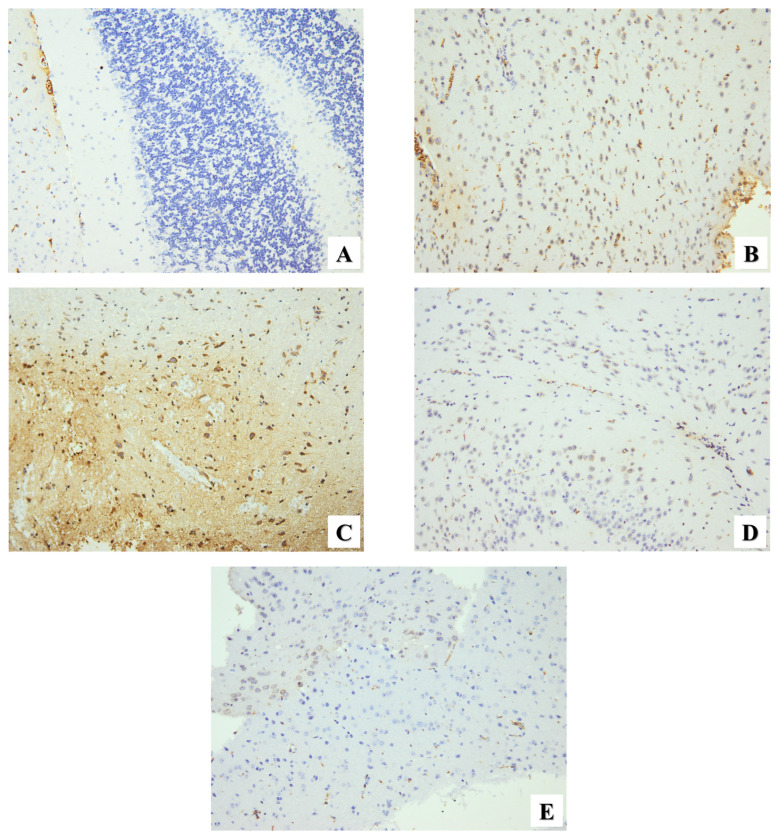
Microscopic immunohistochemical representative images show P53 expression in brain sections of different study groups. (**A**) Normal mice (GI) showing negative P53 expression. (**B**) Infected untreated mice (GII) showing moderate expression. (**C**) Infected mice received PYR+SDZ combination (GIII) showing strong expression. (**D**) Infected mice received ZnO NPs (GIV) showing mild expression. (**E**) Infected mice received Mg-doped ZnO NPs (GV) showing very mild expression. (×200).

For IHC evaluation, the immunohistochemical biomarker P53 was used as an apoptotic indicator in the cerebral tissues of all groups in this experimental study. The healthy group showed no immunoreactivity (Figure 7A). In contrast, the cerebral sections of infected mice showed moderate P53 expression (Figure 7B). A substantial increase in P53 expression was observed in infected mice administered the PYR+SDZ combination (Figure 7C). On the other hand, GIV and GV groups showed a reduction in P53 expression (Figure 7D and Figure 7E, respectively). Contrary to the PYR+SDZ combination, ZnO and Mg-doped ZnO NPs showed reductions in the main immunohistochemical score, particularly Mg-doped ZnO NPs, significantly reducing the immunohistochemical score (Table 4). There is no a significant difference between ZnO- and Mg-doped ZnO NP-treated groups.

**Figure 8:**
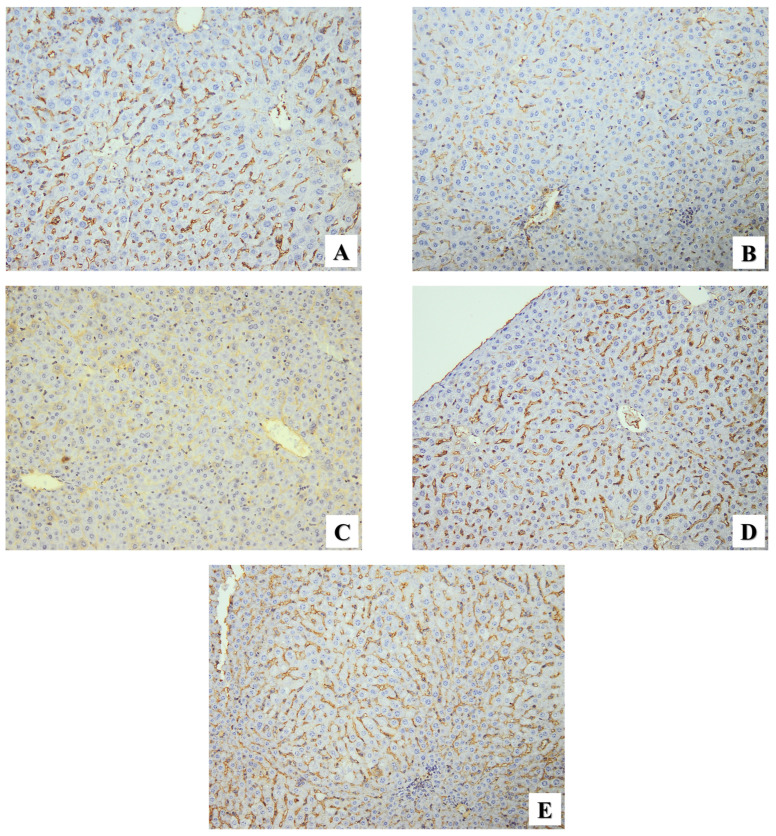
Microscopic immunohistochemical representative images showing CD31 expression in liver sections of different study groups. (**A**) Normal mice (GI) showing positive CD31 expression. (**B**) Infected untreated mice (GII) showing moderate expression. (**C**) Infected mice received PYR+SDZ combination (GIII) showing very mild expression. (**D**) Infected mice received ZnO NPs (GIV) showing strong expression. (**E**) Infected mice received Mg-doped ZnO NPs (GV) showing strong expression. (×200).

In addition, the immunohistochemical biomarker CD31 was used as an angiogenic indicator in the hepatic tissues of all groups in this experimental study. The healthy mice (GI) showed positive CD31 reactivity with liver tissue (Figure 8A). Liver sections of infected mice exhibited moderate CD31 expression (Figure 8B). In comparison to the CD31 level of healthy mice, infected mice administered the PYR+SDZ combination showed very mild expression (Figure 8C), while infected mice treated with ZnO and Mg-doped ZnO NPs showed strong expression (Figure 8D,E respectively) as GI and showed significant increases in the IHS, but without any significant difference between the two groups (Table 4).

## 3. Discussion

Toxoplasmosis is a devastating infectious disease caused by a globally distributed apicomplexan, *T. gondii*. Due to drugs’ inability to pass the blood–brain barrier, which *T. gondii* can do, currently available therapy for chronic toxoplasmosis does not completely eradicate all tissue cysts, increasing the risk of reactivation in immunocompromised patients [34]. Furthermore, PYR-based therapy is associated with adverse reactions and other restrictions as PYR works on the folate biosynthesis pathway, decreasing dihydrofolate reductase activity and ultimately interrupting nucleic acid synthesis [35]. Thus, one of the most intriguing areas of research today is the development of innovative, safe, and efficient treatment solutions for chronic toxoplasmosis. One of the most eye-catching approaches is the application of NPs. Thus, the prospective advantages of employing ZnO and Mg-doped ZnO NPs for treating murine chronic toxoplasmosis were thoroughly investigated in this study by parasitological, histopathological, and immunohistochemical findings.

In the current study, ZnO and Mg-doped ZnO NPs were prepared using a simple chemical co-precipitation approach, and their structural and morphological chrematistics were investigated using XRD, FTIR, XPS, and TEM. The cystogenic ME49 strain of *T. gondii* induced a chronic infection in forty mice, subdivided into four groups of ten mice each. After six weeks, treatment of only three groups of chronically infected mice (GIII, GIV, and GV) was started for 10 days [36]. Mice of GIII received oral PYR (12.5 mg/kg/day) + SDZ (200 mg/kg/day) combination. Mice of GIV and GV received intraperitoneal injections of ZnO NPs (5.6 mg/kg/day) and Mg-doped ZnO NPs (5.6 mg/kg/day), respectively. While spiramycin is frequently used during pregnancy to minimize the teratogenic effects of PYR, the combination of PYR and SDZ is strongly regarded as the optimum therapy against toxoplasmosis [12,13,37]. In the current investigation, GIII did not receive any folinic acid supplements to exclude any potential outside influences on the assessment.

Regarding the characterization of NPs, there were no impurities or extra phases related to the hydroxyl group or unreacted ions in the XRD pattern, suggesting the purity of the synthesized ZnO NPs. Moreover, the composite Mg-doped ZnO showed similar behavior; however, a diffraction peak was observed at 2θ = 43.03° assigned to MgO, confirming the presence of Mg ions in the host ZnO lattice. Further, Mg-doped ZnO showed broadening peaks with low intensity, suggesting a small crystallite size [38,39]. The crystallographic parameters summarized in Table 1 suggest that Mg-doped ZnO NPs have small crystallite sizes, poor crystallinity, and large lattice strain owing to the high Mg^2+^ concentrations (10 wt.%). The large dislocation density of doped ZnO is attributed to crystal defects, a large surface area to volume ratio, and more active sites [39,40]. In general, incorporating Mg ions inside the host ZnO matrix led to structural–morphological modulations. Due to the quantum size effect and the large specific area, Mg-doped ZnO NPs show high carrier transportation and reactive oxygen species, increasing the speed of redox reactions. Furthermore, the tiny nanospheres of Mg-doped ZnO NPs significantly impact the DNA damage, lipid peroxidation, and protein oxidation [18,41].

In the FTIR spectrum, the wavenumber range of 608.85–994.88 cm^−1^ peaks and was attributed to Zn–O stretching modes. This peak increased in the doped ZnO due to Mg–O–Mg stretching bonds. The peak located at 1055 cm^−1^ is associated with the interatomic vibrations of Zn–O, the intensity of which is increased by Mg ion replacements [42]. Additionally, the band at 1404.86 cm^−1^ was attributed to the presence of the hydroxyl group (-OH) resulting from exposure of NPs to the atmosphere. The strong band spotted from 2900 cm^−1^ to 2981 cm^−1^ was ascribed to the C–H stretching vibration. Interestingly, the broadening sharp peaks of the doped samples shifted to a higher wavenumber, clearly revealing the presence of Mg ions in the ZnO framework and increased strain and compressive stress [42,43]. 

In the XPS analysis, the C 1s peak at binding energy 285.07 eV was ascribed to the carbon element as a reference in the XPS instrument. Mg-doped ZnO showed a strong peak at 1304.43 eV related to the presence of Mg in the spectrum. The high spin–orbit interaction resulted in the splitting of Zn 2p states. The deconvoluted O 1s spectrum with a sliding shift to higher binding energy is related to the presence of defects or oxygen vacancies, which is useful in the biotechnological field [44,45]. The obtained results from the FR-IR and XPS analyses emphasize the integration of Mg^2+^ into the ZnO framework.

The average size and particle distribution were visualized using TEM micrographs; the ZnO nanosphere appears not uniform and aggregated together, and the surface is not homogeneous. These observations agree with that recently reported by Hessien [46]. Mg-doped ZnO showed smaller nanosphere diameters (~23 nm), with high density attributed to the large surface area. As demonstrated, some of the particles possess a tiny spherical shape of ~12 nm that is in agreement with previous studies [46,47]. Moreover, as recently mentioned, the presence of Mg ions prevents the aggregation of particles, in addition to size reduction, which indicates the high density on the surface and high chemical reactivity of Mg-doped ZnO [41,48].

Regarding the parasite burden, the intraperitoneal injection of ZnO and Mg-doped ZnO NPs statistically caused a significant reduction (*p* < 0.05) in the tissue cyst numbers (29.30% and 35.08%, respectively). In comparison, the PYR+SDZ combination was more effective and showed a significant reduction (*p* < 0.05) (71.41%) compared to the chronically infected group. This finding is consistent with the results of Mohammad et al. [49], who found that the brain cyst count in infected mice treated with Cu-BTC MOF was significantly reduced by 24.4%. Moreover, in consensus, Saadatmand et al. [30] revealed that the oral administration of Zn NPs for 14 days at doses of 32.5 and 75 mg/kg/day significantly decreased (*p* < 0.001) the mean number and diameter of the brain tissue cysts. In the same manner, Khashan et al. [22] observed that the synthesized Al-doped ZnO NPs demonstrated cytotoxic efficacy with high reduction values reaching 64.5 and 48% against *Leishmania tropica* and *L. donovani*, respectively, in addition to high antibacterial activity and promising anticancer activity. This cytotoxic effect of NPs could be highly relevant to generating reactive oxygen species (ROS) and releasing zinc ions, which enhance cellular permeability and have toxic consequences [22,50].

For a more comprehensive evaluation, all experimental groups’ tissue sections from the brain, liver, and spleen were examined. The histopathological examination of brain tissue sections showed a marked ameliorative impact of the ZnO and Mg-doped ZnO NP administration on the tissue cysts and tissue abnormalities such as encephalitis, gliosis, brain edema, neurodegeneration, and inflammatory cell infiltration. Moreover, an examination of liver sections from chronically infected mice showed severe histopathological necrotic changes such as hydropic degeneration, ballooning hepatocytes, and mononuclear inflammatory cells. These tissue abnormalities were significantly reduced after treatment with ZnO and Mg-doped ZnO NPs. The spleen sections of the infected group showed lymphocytic depletion of splenic lymphoid follicles and frequent deposition of hemosiderin pigment. The presence of hemosiderin pigment may be a sign of hemolytic anemia and persistent congestion due to macrophages engulfing RBCs [51]. The splenic tissue of the infected mice treated with ZnO and Mg-doped ZnO NPs showed near-normal histological architecture with congested blood vessels and few hemosiderin pigment depositions.

These results concur with a previous study revealing that inflammation induced by chronic toxoplasmosis infection in the brain was significantly decreased after treatment with mefloquine niosomes [52]. Etewa et al. [53] revealed that the brain, liver, and spleen histopathological images were significantly improved in infected mice treated with spiramycin-loaded chitosan NPs compared to other groups. Furthermore, the brain tissue of mice infected with schistosomiasis responded favorably to an intraperitoneal injection of 5.6 mg/kg/day ZnO NPs for five days [54]. In contrast, infected mice treated with kumquat extract-loaded chitosan NPs showed severe pathological changes in the brain [55]. Although a previous study [32] stated that the doping of Mg ions enhanced the antibacterial efficacy on ZnO NPs, there is no significant difference between the administration of ZnO or Mg-doped ZnO NPs.

Moreover, *Toxoplasma* infection regulates many cellular pathways to support its survival and proliferation. Many previous studies reported that the activation of P53 is associated with pathological changes in the cell, such as cell cycle arrest, oxidative stress, and DNA damage [8,9,56]. The important role of p53 in parasitic infectious diseases has been reported in previous studies [57,58]. As *T. gondii* intracellular invasion affects the process of genetically programmed cell death, P53 expression was used to evaluate brain tissue apoptosis. However, our findings in the brain sections of infected mice show moderate P53 expression. A previous study has revealed that the parasite inhibited the apoptosis pathway in the infected cells during the chronic phase of toxoplasmosis [59]. According to earlier studies, inhibiting apoptosis in the infected neuronal cells can be an easy way to survive and bypass the host’s immunological defense [60,61]. However, PYR+SDZ combination-treated mice showed strong P53 expression, and El Sharazly et al. have denied the correlation between PYR+SDZ efficacy and its induced apoptosis as the mean number of TUNEL-positive cells in brain sections was low [52]. ZnO- and Mg-doped ZnO NP-treated mice showed mild P53 expression that indicated the protective effects of ZnO and Mg-doped ZnO NPs against apoptosis in the cerebral tissue [62]. The anti-apoptotic action of ZnO NPs has been mentioned in earlier research that linked its protective and anti-apoptotic effect to its antioxidant activity that minimizes the stress on living cells [27,63].

The IHC biomarker CD31 was also used as an angiogenic indicator in the hepatic tissues. While infected mice showed moderate immunoreactivity with CD31, the PYR+SDZ combination showed mild expression. On the other hand, infected mice treated with ZnO showed strong expression of CD31, while Mg-doped ZnO NPs showed the same expression in healthy mice. In agreement with our results, Dincel and Atmaca have reported that toxoplasmosis infection-induced oxidative stress can reduce angiogenesis [64]. Recently, metal oxide nanoparticles were recommended to promote angiogenesis because of their well-known ability to generate ROS [65]. So, our findings indicate the restoration of balance between free radicals and antioxidant defense activity [64].

## 4. Materials and Methods

### 4.1. Ethics Proclamation

The Institutional Animal Care and Use Committee (IACUC) of Zagazig University, Egypt approved this research study procedure following international animal care standards, including therapy and euthanasia (ZU-IACUC/1/F/78/2022).

### 4.2. Synthesis of NPs

#### 4.2.1. Materials for Synthesis of NPs

The used materials were zinc acetate dihydrate Zn (CH_3_COO)_2_·2H_2_O ≥ 98.0%), magnesium chloride hexahydrate (MgCl_2_·6H_2_O, 98.0%, extra pure), sodium hydroxide (NaOH, ≥98.0%), acetone, and isopropanol. Chemicals and reagents were purchased from Merck and Alfa Aesar company, Karlsruhe, Germany.

#### 4.2.2. Synthesis of Un-Doped ZnO and Mg-Doped ZnO Using Co-Precipitation Method

Zinc oxide nanopowder was prepared by dissolving 6.68g of Zn (CH_3_COO)_2_·2H_2_O in 30 mL of deionized water under a magnetic stirrer for 2 h. Sodium hydroxide solution was prepared by dissolving 2.47 g of NaOH in 30 mL of deionized water for 30 min. NaOH solution was carefully added to zinc acetate solution to form the precipitate powder with continuous stirring for 5 h at room temperature. The resulting white precipitate powder was obtained at a pH reaction equal to 9. The resulting white powder was separated using filter paper and washed many times with double-distilled water to remove residuals or undesired salts. After that, the product precipitate was dried and calcinated at 400 °C for 2 h in an oven. Magnesium-doped zinc oxide (Mg_x_Zn_1−x_O, x = 10 wt.%) nanocomposite was prepared by dissolving 0.87 g MgCl_2_·6H_2_O in 10 mL deionized water, then added to 4.50 g of Zn (CH_3_COO)_2_·2H_2_O in 40 mL deionized water under stirring for 3 h. NaOH solution (mmm) was added drop by drop to the mixture until the precipitate was formed at a pH reaction equal to 9. The product precipitate powder was filtered, washed, dried, and eventually annealed at the same previous conditions.

### 4.3. Characterization of Un-Doped ZnO and Mg-Doped ZnO

The crystal structure and crystallographic parameters of un-doped ZnO and Mg-doped ZnO were characterized by using X-ray diffraction analysis (XRD) (Bruker D8 Advance Eco; Bruker AXS, Karlsruhe, Germany), working at λ = 1.54 A (Cu Kα radiation) with 2θ angle varied from 30° to 80°. Fourier transform infrared (FTIR spectrum 100, Perkin Elmer) was employed to determine the functional group and molecular interaction of NPs. X-ray photoelectron spectroscopy (XPS) analysis was examined to identify the nanomaterials’ chemical state and electronic structure. Transmission electron microscopy (TEM; Hitachi-H-7500; Hitachi, Tokyo, Japan) was performed for further microstructural analysis.

### 4.4. Experimental Design

#### 4.4.1. Parasite Strain and Infection

Theodore Bilharzia Research Institute (TBRI), Giza, Egypt, donated the brain suspension of a mouse chronically infected with the cystogenic ME49 strain of *T. gondii.* Then, the brain suspension was diluted by sterile phosphate-buffered saline (PBS) to a concentration of 100 cysts per milliliter (mL). Finally, 0.1 mL of this diluted suspension containing 20 cysts was used to infect each mouse [66].

#### 4.4.2. Animal Model

This experimental case–control study was conducted at the Medical Parasitology Department, Faculty of Medicine, Zagazig University, Egypt. For our experiment, fifty male Swiss albino lab mice, six to seven weeks old, were used. All test mice were carefully maintained in well-ventilated cages with access to water and daily grain feeding. Furthermore, under meticulously monitored lighting (12 h of light and 12 h of darkness), the laboratory’s temperature was maintained at 25 ± 2 °C.

#### 4.4.3. Treatment Schedule

Five experimental groups, each with ten mice, were created by randomly selecting all the animals. The mice in the first group (GI: healthy group) were healthy and infection-free, while those in the other four groups received 0.1 mL of brain suspension orally to establish chronic toxoplasmosis. The third, fourth, and fifth groups (GIII, GIV, and GV) began receiving treatment day by day for 10 days after six weeks of infection [36] as follows:

GII: infected without any treatment group.
GIII: orally received PYR (12.5 mg/kg/day) + SDZ (200 mg/kg/day) combination [67].
GIV: intraperitoneally administered ZnO NPs (5.6 mg/kg/day) [54].
GV: intraperitoneally administered Mg-doped ZnO NPs (5.6 mg/kg/day).

At the end of the experiment, all mice were sacrificed according to international animal care standards.

#### 4.4.4. Collecting Samples

After sacrifice, the right hemisphere of each mouse’s brain was used to estimate the existence of parasite tissue cysts, while the left hemisphere, liver, and spleen of each mouse were collected and preserved in labeled containers with appropriate volumes of 10% formalin for fixation to perform further histopathological and immunohistochemistry (IHC) evaluations.

### 4.5. Parasitological Assessment

#### 4.5.1. Parasitic Burden Assessment

For preparation of the brain suspension, the right brain hemisphere of each mouse was homogenized in an equal volume of PBS using a tissue homogenizer for 5 min. One drop (10 µL) of the brain homogenate was dropped on a sterile microscopic glass slide, and then the tissue cyst burden was quantified under ×400 magnification using a light microscope [68]. Finally, the total count of the brain cysts was calculated for each mouse and, subsequently, for each group.

#### 4.5.2. Histopathological Assessment

Fixation and tissue processing, as well as hematoxylin and eosin (H&E) staining of brains, livers, and spleens, were carried out [69]. Histopathological analysis was performed using conventional light microscopy.

#### 4.5.3. Immunohistochemistry Assessment

For IHC assessment, 4 µm thick sections of brain and liver from different animal groups that were formalin-fixed and paraffin-embedded were prepared using a microtome and placed on saline-coated microscopic glass slides, which were then left overnight for incubation at room temperature. The paraffinized sections were dewaxed in xylene and rehydrated to water via ethanol in graded concentrations. The endogenous peroxidase activity was eliminated with H_2_O_2_ in methanol, and then sections were pre-treated in sodium citrate buffer in a microwave. The slides were washed three times in tris-buffered saline after being allowed to cool for 15 min [70]. Pre-prepared slides for brains and livers were incubated with anti-P53 (1:8000; Santa Cruz Biotechnology; Santa Cruz, CA, USA) and anti-CD31 (1:100; Novus; Littleton, CO, USA) antibodies for a night at 37 °C. On the next day, secondary and tertiary antibodies were used on each section for 90 min. All sections were treated with chromogen diaminobenzidine tetrachloride (DAB), counterstained with hematoxylin, dehydrated, cleaned, and mounted. Finally, IHC sections were examined using a light microscope at different magnifications.

#### 4.5.4. Inflammatory and Immunohistochemical Scoring

The inflammatory score (IS) was calculated [71] and modified. In essence, tissues were examined using a 40× objective in a blinded way and rated as follows: 0 = no inflammation, 1 = a few inflammatory cells, 2 = perivascular inflammation infiltration, and 3 = perivascular cuffing intensity spread to surrounding tissues. Two researchers performed the score evaluation. The immunohistochemical score (IHS) of each P53 and CD31 was calculated by multiplying the percentage of positive staining cells by the intensity degree of the stain and ranged from 0 (no immunoreactivity) to 12 (maximum immunoreactivity) [56]. The staining percentage was classified as follows: 0 = 0%, 1 = <10%, 2 = 10–25%, 3 = 26–50%, and 4 = 51–100%. The staining intensity was classified as follows: 0 = none, 1 = weak, 2 = moderate, and 3 = strong. Finally, IHS was classified as follows: negative immunoreactivity (0) = 0, weak (1–4) = 1, moderate (5–8) = 2, and strong (9–12) = 3.

### 4.6. Analytical Statistics

The data are provided as the mean ± standard deviation (SD). Statistical Package for the Social Sciences (SPSS) version 23.0 was used for the one-way analysis of variance (ANOVA) test and statistical analysis. Tukey’s post hoc tests were used for various comparison analyses between study groups. The Kruskal–Wallis (*H*) test, pairwise comparison, was used for histopathological and immunohistochemical scoring, followed by Dunn’s post hoc test. When *p*-values were less than 0.05, group differences were deemed statistically significant [72].

## 5. Conclusions

In this experimental study, the XRD pattern revealed that Mg-doped ZnO NPs are of weak crystallinity and a small crystallite size. FTIR and XPS analyses confirmed the integration of Mg ions into the ZnO framework, producing the high-purity Mg-doped ZnO nanocomposite. In addition, the TEM micrographs determined the particle size of un-doped ZnO in the range of 29 nm, which was reduced to around 23 nm with Mg^2+^ replacements. The microstructure analysis results demonstrate that Mg-doped ZnO NPs possess a small particle size and large surface area. The parasitological findings reveal that treatment with ZnO and Mg-doped ZnO NPs statistically reduced tissue cyst numbers and improved *T. gondii*-induced adverse repercussions in the brain, liver, and spleen. Additionally, NPs could inhibit tissue apoptosis and activate angiogenesis. Before starting clinical trials. More laboratory investigations should be conducted to assess the potential effects of utilizing ZnO and Mg-doped ZnO NPs against the other pathways and tissues.

## Figures and Tables

**Table 1:** XRD parameters of ZnO and Mg-doped ZnO NPs.

Nanoparticles	D (nm)	$\varepsilon \times 10^{-3}$	$\delta \times 10^{-4}{\left(nm\right)}^{-2}$	$X_{c}$
ZnO	29.00	1.05	12.00	52.00
Mg-doped ZnO (10 wt.%)	23.00	1.52	20.00	41.00

**Table 2:** *T. gondii* brain cyst main burden in differently treated groups compared to the infected untreated group.

Groups	Tissue Cysts (Mean ± SD)	Std. Error	Reduction (%)	*F*	*p*-Value
GII	4205.00 ± 136.32	43.11	-	1036.82	<0.001 *
GIII	1202.00 ± 107.48	33.99	71.41		
GIV	2973.00 ± 94.64	29.93	29.30		
GV	2730.00 ± 139.84	44.22	35.08		

* Statistically significant at *p*-values ≤ 0.05.

**Table 3:** The mean inflammation score (IS) in brain, liver, and spleen sections of experimental groups.

Groups	IS in Brain (mean ± SD)	IS in Liver (mean ± SD)	IS in Spleen (mean ± SD)
GI	0.00 ± 0.00	0.00 ± 0.00	0.00 ± 0.00
GII	2.50 ± 0.71	2.85 ± 0.42	2.60 ± 0.70
GIII	2.65 ± 0.70	2.70 ± 0.48	2.70 ± 0.68
GIV	0.70 ^a^ ± 0.68	1.00 ^a^ ± 0.94	1.20 ± 1.23
GV	0.50 ^a^ ± 0.71	0.80 ^a^ ± 0.79	1.20 ± 1.14
*H*	37.26	37.62	29.75
*p*-value	<0.001 *	<0.001 *	<0.001 *

*H* is the Kruskal–Wallis test statistic. Dunn’s post hoc test was used to perform multiple comparisons between groups. * Statistically significant at *p*-values ≤ 0.05. ^a^ Significant with GII (Infected non-treated group).

**Table 4:** The mean immunohistochemical score (IHS) of P53 and CD31 in the brains and livers of experimental groups, respectively.

Groups	IHS of P53 (mean ± SD)	IHS of CD31 (mean ± SD)
GI	0.00 ± 0.00	3.00 ± 0.00
GII	1.80 ± 0.63	1.60 ± 0.52
GIII	2.70 ± 0.48	0.70 ± 0.48
GIV	0.50 ± 0.53	2.70 ^a^ ± 0.48
GV	0.40 ^a^ ± 0.67	2.80 ^a^ ± 0.42
*H*	38.49	40.23
*p*-value	<0.001 *	<0.001 *

*H* is the Kruskal–Wallis test statistic. Dunn’s post hoc test was used to perform multiple comparisons between groups. * Statistically significant at *p*-values ≤ 0.05. ^a^ Significant with GII (Infected non-treated group).

## Data Availability

The data presented in this study are available on request from the corresponding author.
